# Validating a set of tools designed to assess the perceived quality of training of pediatric residency programs

**DOI:** 10.1186/s13052-014-0106-2

**Published:** 2015-01-20

**Authors:** Liviana Da Dalt, Pasquale Anselmi, Sara Furlan, Silvia Carraro, Eugenio Baraldi, Egidio Robusto, Giorgio Perilongo

**Affiliations:** Paediatric Residency Program, Department of Woman’s and Child’s Health, University of Padua, Via Giustiniani 3 – 35128, Padua, Italy; Department FISPPA, University of Padua, Padua, Italy

**Keywords:** Evaluation tools, Validation study, Many-facet Rasch model, Pediatric residency program, Teaching faculty

## Abstract

**Background:**

The Paediatric Residency Program (PRP) of Padua, Italy, developed a set of questionnaires to assess the quality of the training provided by each faculty member, the quality of the professional experience the residents experienced during the various rotations and the functioning of the Resident Affair Committee (RAC), named respectively: “Tutor Assessment Questionnaire” (TAQ), “Rotation Assessment Questionnaire” (RAQ), and RAC Assessment Questionnaire”. The process that brought to their validation are herein presented.

**Method:**

Between July 2012 and July 2013, 51 residents evaluated 26 tutors through the TAQ, and 25 rotations through the RAQ. Forty-eight residents filled the RAC Assessment Questionnaire. The three questionnaires were validated through a many-facet Rasch measurement analysis.

**Results:**

In their final form, the questionnaires produced measures that were valid, reliable, unidimensional, and free from gender biases. TAQ and RAQ distinguished tutors and rotations into 5–6 levels of different quality and effectiveness. The three questionnaires allowed the identification of strengths and weaknesses of tutors, rotations, and RAC. The agreement observed among judges was coherent to the predicted values, suggesting that no particular training is required for developing a shared interpretation of the items.

**Conclusions:**

The work herein presented serves to enrich the armamentarium of tools that resident medical programs can use to monitor their functioning. A larger application of these tools will serve to consolidate and refine further the results presented.

**Electronic supplementary material:**

The online version of this article (doi:10.1186/s13052-014-0106-2) contains supplementary material, which is available to authorized users.

## Introduction

The fundamental issue of monitoring and evaluating the quality of training provided by accredited post-graduate medical training programs remains widely unsolved, at least in Europe. A variety of components contribute to the quality of the training; thus, a comprehensive articulated multi-dimensional system should be conceived to assess it. Periodic, “proxy-evaluations” by an independent third party is the inspiring model adopted by those countries in which the issue has been or is going to be addressed [1]. Concurrently, tools to evaluate at least some of the components determining the quality of the training, notably, the teaching performance of the faculty, have been validated and largely developed [2-9].

The Paediatric Residency Program (PRP) of the University of Padua, in Italy, while waiting for having the proxy-evaluation system implemented, addressed the issue of monitoring and evaluating the quality of the training by having the program going through a periodic, systematic ISO:9001 certification process [10]. Furthermore, it implemented a comprehensive evaluation system [11]. This system targets the following components: i) the residents’ knowledge and performance; ii) the quality of the teaching provided by the Faculty, iii) the quality of professional experience maturated during each rotation they go though and iv) of the functioning of the Resident Affair Committee (RAC), the body in charge of running the program. The “In-training examination”, provided by the American Board of Paediatrics, is the tool adopted to evaluate the residents’ knowledge [12], while, for evaluating all the other components of the program, a series of web-based ad hoc questionnaires has been developed.

Herein, we describe the results of the validation process of the questionnaires, which were elaborated to evaluate the quality of teaching provided by the Faculty, the rotations and the functioning of the RAC: named respectively the “Tutor Assessment Questionnaire” (TAQ), the “Rotation Assessment Questionnaire” (RAQ), and the “RAC Assessment Questionnaire”. The validation of these tools is a fundamental pre-requisite to implement their use. The validation of the questionnaire elaborated to evaluate residents’ performance has been already published [11].

## Methods

The PRP of the University of Padua is a 5-year national accredited program for post-graduate training in Pediatrics. Approximately 80% of learning activities takes place in the clinical setting, practicing medicine under faculty’s supervision with the ultimate goal of increasing the levels of responsibilities throughout training. The remaining learning activities include formal lectures (e.g. ground rounds and ward lectures), seminars, workshops and personal studies. Residents rotate through 15 of the 25 Divisions/Services of the Department of Woman’s and Child’s Health of Padua and of the affiliated Hospitals during their first three years; rotations range in time from three to six months. During the last two years of training, residents select elective rotations involving at most three divisions, each lasting from six to twelve months. The “tutors” under evaluation by the residents were the senior members of the Faculty in charge of running the service and of organizing and supervising the activity of the residents. Tutors have also the responsibility of providing the residents initiating the rotation with clear information regarding the learning objectives and the way the service is organised and the rotation planned. The RAC is composed by the Program Director, three faculties and the chief resident. It has the ultimate responsibility of running the PRP.

The three questionnaires, TAQ, RAQ and the RAC Assessment Questionnaire, consist of 9, 12, and 15 items respectively. They were constructed in order to be easy to complete and minimally time consuming. Participants rated each items on a five-point scale from 1 (“poor”) to 5 (“excellent”; see Additional file 1); the last item of each questionnaire asked for an overall comprehensive judgment. The residents were given brief instructions about completing the questionnaires.

In Italy, the academic year for residents goes from July to June. The data used for validating the three questionnaires were collected between July 2012 and July 2013. Residents completed the TAQ and the RAQ within two weeks by the end of each rotation and, the RAC Assessment Questionnaire, yearly, within two weeks by the end of the academic year. Albeit the questionnaires were not anonymous, it was up to the RAC to ensure that the individual resident was unrecognizable when providing feed-backs to the Faculty.

A many-facet Rasch measurement (MFRM, [13]) analysis was used for the validation of the questionnaires. Peculiar features of Rasch models (e.g., transformation of ordinal raw scores into interval measures, identification of poorly functioning items, reproducibility of results across samples and items, investigation of response behavior) make them valuable tools in both the analysis of clinical data [14-17], and the development and evaluation of instruments [18-21].

### Many-Facet Rasch Measurement (MFRM)

The MFRM [13] is a formal model for transforming nonlinear, scale-dependent ordinal raw scores into linear, scale-free interval measures. In its basic form, the model represents the probability *P*_*nijk*_ of an examined *n* being given by judge *j* a score *k* on an item *i* as an effect of the ability of examined *n* (β_*n*_), the difficulty of item *i* (δ_*i*_), the severity of judge *j* (γ_*j*_), and the impediment in giving a score *k* rather than *k*-1 (τ_*k*_):

Examined, judges, and items are facets. In the analyses that follow, the residents are the judges and, according to the questionnaire under consideration, the tutors, the rotations or the RAC are the examined. Facets concerning the gender of residents (ε), and the year in the program (ζ) are considered as well.

The MFRM analyses are performed using the computer program Facets 3.71.3 [22]. A measure is computed for each element of each facet. Greater measures mean more positive evaluations for examined, greater difficulty (i.e., fewer positive evaluations) for items, and greater severity for judges (residents).

The validation of each of the three questionnaires has been conducted by taking into account aspects concerning the functioning of the items and that of the response scale, the dimensionality, reliability and construct validity of the questionnaire [11].

The functioning of the items is assessed using item mean square fit statistics (infit and outfit). Values greater than 1.4 suggest that the item degrades the measurement system, or that it assesses a construct that is different from the principal one being measured (Rasch dimension) [23]. Principal component analyses of standardized residuals are also run, where contrasts in the residuals with values greater than 3 are indicative of multidimensionality [24].

The functioning of the response scale is assessed by determining whether the step calibrations τ_*k*_ are ordered or not. If they are not ordered, the response scale is not adequate for the measurement purpose [25]: for instance, the highest rating scale categories are not the most probable ones for the examined with the highest levels of performance.

Reliability of each questionnaire is assessed by examining the spread of examined measures on the latent variable. Internal consistency is assessed by means of the indexes separation reliability (*R*) and strata of examined. When there are not missing data, *R* is the Rasch equivalent of Cronbach’s α [26]. Strata evaluates the number of statistically distinct groups of examined that the questionnaire is able to discern [27]. For instance, Strata = 2 means that the questionnaire is able to discern the best examined from the worst. Inter-rater reliability is assessed by comparing the observed percentage of agreement among judges with that expected when their different degrees of severity are taken into account.

Construct validity of each questionnaire is assessed by examining the spread of the item difficulties along the latent variable. In particular, the item strata identify the number of statistically distinct groups of item difficulties that the judges can discern. In addition, bias interaction analyses are performed in order to investigate whether the functioning of the items differs with the gender of judges. The first test provides information about content representativeness, whereas the second test provides information about construct reproducibility [26-28].

It is a requirement of the Italian Law on Post-graduate Medical Residency Programs to set evaluation system. As a such, the study presented was not considered a research proposal to be approved by Ethical Committee. However, we submitted our Comprehensive evaluation program to the Institutional Review Board of our University and we received an informal approval.

## Results

### Validation of the Tutor Assessment Questionnaire (TAQ)

Fifty-one residents (47 females, No. 14, 14, 10, 9, 4 for 1st to 5th year residents, respectively) evaluated 26 tutors. Each resident evaluated from 1 to 6 tutors, and each tutor received from 1 to 16 evaluations. Given the longer duration of rotations, a smaller number of evaluations was provided by the residents of the last two years. The data matrix had dimensions 159 (overall number of evaluations) × 9 (items).

The functioning of Item 1 (“[The tutor] respects time tables of the collective activities) changed with the gender of residents. In particular, male residents provided more severe evaluations than female residents (*t*(19) = 2.17, *p* < .05). A new analysis was run without item 1. The remaining 8 items defined a substantively unidimensional scale (the first contrast in the residuals had a value of 2), and none of them exhibited misfit.

The step calibrations were ordered (τ_poor-mediocre_ = − 1.04; τ_mediocre-respectable_ = − .84; τ_respectable-good_ = − .19; τ_good-excellent_ = 2.07). Therefore, residents used adequately the response scale.

Locations of residents, tutors and items on the latent variable are depicted as below (see Table 1).

**Table 1:** **Validation of the Tutor Assessment Questionnaire (TAQ) - Locations of residents, tutors and items on the latent variable**

**Measure**	**Resident**	**Tutor**	**Item**
4		T13	
3		T23	
		T9	
2			
		T21	
	R50		
	R9		
	R21	T24	
1		T3	[Encourages performing invasive procedures]
	R36	T27 T11	
	R16		[Regularly gives ward lectures]
	R28 R27	T22	
	R32 R51 R1		
0	R30	T25	[Reviews clinical documentation] [Scrutinizes clinical problems]
	R5 R44	T20 T17 T26 T12	[Effective in conducting lectures]
	R45 R11 R22 R24 R52	T18 T10	[Overall judgment] [Promotes self-education]
	R46 R48	T4 T7	[Promotes autonomy and accountability ]
	R37 R8 R26 R10 R29 R3	T8	
-1	R31 R2 R43 R34 R49	T15 T5 T6	
	R6 R7 R4 R23	T2 T16	
	R15 R40 R14 R18	T14	
	R47 R33 R13 R42	T1	
-2			
	R38		
	R17		
	R20		
-3	R12		
	R19		
-4	R39		
	R35		
-5	R41		

Greater measures for residents indicate that they were more severe; greater measures for tutors indicate that they received more positive evaluations, and greater measures for items indicate that they were more difficult. Table 2 reports the summary statistics for the eight items. Overall, tutors received more positive evaluations on Item 7 (“[The tutor] promotes autonomy and accountability”) and less positive evaluations on Item 8 (“[The tutor] encourages performing invasive procedures”). These eight items allowed the residents to identify almost seven levels of different quality (strata = 6.93). Moreover, they provided a reliable evaluation of tutors (*R* = .92), and allowed the distinction of tutors in almost five levels.

**Table 2:** **Average scores, item measures, standard errors and fit statistics of the Tutor Assessment Questionnaire (measure order)**

**Item**	**Average score**	**Measure**	***SE***	**Infit**	**Outfit**
8 [Encouragesinvasive procedures]	2.8	.98	.09	1.30	1.21
2 [Regularly gives ward lectures]	3.1	.58	.09	1.19	1.29
6 [Reviews clinical documentation]	3.5	.05	.10	1.05	1.09
4 [Scrutinizes clinical problems]	3.5	-.05	.10	.87	.86
3 [Is effective in conducting lectures]	3.7	-.23	.10	1.16	1.16
9 [Overall judgment]	3.7	-.36	.10	.51	.52
5 [Promotes self-education]	3.7	-.37	.10	.70	.74
7 [Promotesautonomy and accountability]	3.9	-.60	.11	1.02	1.15

*Note*. Greater measures indicate more difficult items (i.e., that received fewer positive evaluations).

Although residents differed in severity (χ^2^(50) = 645.4, *p* < .001), the observed agreement among them is in line with that expected (Exp = 28.4%; Obs = 29%). The severity of residents did not change with their gender (χ^2^(1) = .6, *p* = .45), but with the year of program that they were attending (χ^2^(4) = 26.6, *p* < .001).

### Validation of the Rotation Assessment Questionnaire (RAQ)

Fifty-one residents (46 females; No. 14, 14, 10, 9, 4 for 1st to 5st year residents, respectively) evaluated 25 rotations. Each resident evaluated from 1 to 7 rotations, and each rotation received from 1 to 16 evaluations. The data matrix had dimensions 164 (overall number of evaluations) × 12 (items).

The value of the first contrast in the residuals was 2.9, very close to the criterion indicative of multidimensionality. In addition, Item 3 (“rotation was organized as declared”), Item 4 (“Teaching activities were regularly delivered”), and Item 11 (“[The rotation] allowed participation in other cultural activities of the program”) exhibited misfit (infit = 1.43, outfit = 1.52 for Item 3; infit = 1.46, outfit = 1.51 for Item 4; infit = 1.85, outfit = 2.09 for Item 11). These items were excluded and a new analysis was run. The infit of Item 7 (“[The rotation] contributed to improve my clinical skills”) exceeded the criterion of 1.4. However, its value (1.47) is not so large to require the removal of the item from the pool. The 9 items defined a substantively unidimensional scale (the value of the first contrast decreased from 2.9 to 2.1), and none of them exhibited differential gender functioning.

The step calibrations were ordered (τ_poor-mediocre_ = − 1.42; τ_mediocre-respectable_ = − 1.15; τ_respectable-good_ = − .05; τ_good-excellent_ = 2.61). Therefore, the response scale had been adequately used by residents.

Locations of residents, rotations and items on the latent variable are depicted as below (see Table 3).

**Table 3:** **Validation of the Rotation Assessment Questionnaire (RAQ) - locations of residents, rotations and items on the latent variable**

**Measure**	**Resident**	**Rotation**	**Item**
5			
		RO23	
4			
3			
			[It contributed to improve clinical skills]
2		RO21 RO22	
	R50		
		RO3	
1	R9	RO24	
		RO9	
	R5	RO6	
	R28	RO26 RO20 RO7 RO25	[A clear definition of learning objectives provided] [Learning objectives met]
0	R45	RO4 RO27	
	R16 R27	RO10	[Overall judgment] [It served to improve professional competences]
	R32 R52	RO8	[It contributed to improves pediatric knowledge] [It encouraged personal studies]
	R14	RO19 RO17	[It has been an educational experience] [It has been an enriching experience from a human point of view]
	R21 R11 R10		
-1	R30 R24 R26	RO18 RO12	
	R29 R36	RO5 RO16	
	R3 R43 R44 R51 R1 R18 R6		
	R48 R49	RO15 RO1	
	R8	RO2	
-2	R47 R46 R22 R33	RO14	
	R23 R7 R2 R37 R4		
	R34 R20 R13		
	R40		
	R15		
-3	R12 R31		
	R42		
	R25		
	R38 R17		
	R35		
-4			
-5	R39 R19		

Greater measures for rotations indicate that they received more positive evaluations. The measures of residents and items read as in Table 1. Table 4 contains summary statistics for the 9 items. On the whole, the rotations received more positive evaluations on Item 9 (“[The rotation] has been a enriching experience from a human point of view”) and less positive evaluations on Item 7 (“[The rotation] contributed to improve my clinical skills”). These 9 items allowed the residents to distinguish ten qualitatively different levels in the rotation quality (strata = 10.02). In addition, they provided a reliable evaluation of rotations (*R* = .95), and allowed to distinguish them in almost six groups of different quality (strata = 5.88).

**Table 4:** **Average scores, item measures, standard errors and fit statistics of the Rotation Assessment Questionnaire (measure order)**

**Item**	**Average score**	**Measure**	***SE***	**Infit**	**Outfit**
7 [It contributed to improve my clinical skills]	2.4	2.20	.10	1.47	1.33
1 [Definition of learning objectives]	3.6	.26	.11	.92	1.15
2 [The learning objectives have been met]	3.6	.18	.11	.76	1.03
12 [Overall judgment]	3.8	-.22	.11	.68	.73
6 [It improved professional competences]	3.9	-.27	.11	.75	.84
5 [It contributed to improve pediatric Knowledge]	4.0	-.47	.12	.74	.73
10 [It encourages personal study]	4.0	-.49	.12	.98	1.08
8 [It has been an educational experience]	4.0	-.59	.12	.85	.86
9 [It has been a human enriching experience]	4.0	-.60	.12	1.38	1.29

*Note*. Greater measures indicate more difficult items (i.e., that received fewer positive evaluations).

The residents differed in severity (χ^2^(50) = 749.8, *p* < .001), but the observed agreement among them is in line with that expected (Exp = 34.6%; Obs = 34.9%). Male and female residents did not differ in severity (χ^2^(1) = 1.0, *p* = .31). There were some differences among residents attending different years of program, but they were not reliable (χ^2^(4) = 16.6, *p* < .01; *R* = .35).

### Validation of the RAC Assessment Questionnaire (RACAQ)

Forty-eight residents (42 females; N = 14, 13, 8, 11, 2 for 1st to 5th year residents, respectively) evaluated the RAC. The data matrix had dimensions 48 (evaluations) × 15 (items).

The functioning of Item 14 (Scoring “the teaching attitude of the faculty”) changed with the gender of residents (i.e., males provided more severe evaluations than females; *t*(7) = 2.38, *p* < .05), whereas Item 5 (“[The RAC] It provides residents with individualized feedbacks regarding the evaluation they received”) exhibited misfit (infit = 1.64, outfit = 1.66). These two items were excluded and a new analysis was run. The remaining 13 items defined a substantively unidimensional scale (the value of the first contrast in the residuals was equal to 2.1) and none of them exhibited misfit (the largest Infit was 1.41).

The step calibrations were ordered (τ_poor-mediocre_ = − 3.08; τ_mediocre-respectable_ = − 1.81; τ_respectable-good_ = .48; τ_good-excellent_ = 4.40). Therefore, residents used the response scale appropriately.

Locations of residents and items on the latent variable are depicted as below (see Table 5), whereas Table 6 contains summary statistics for the items. On the whole, the RAC received more positive evaluations on Item 10 (“Scoring the capacity of the program to promote clinical autonomy and reliability”) and less positive evaluations on Item 4 (“[The RAC] cares of individual residents”). These measures provided a reliable depiction of strengths and weaknesses of the RAC (*R* = .95; Strata = 6.14).

**Table 5:** **Validation of the RAC Assessment Questionnaire - Locations of residents and items on the latent variable**

**Measure**	**Resident**	**Item**
3		
	R25	
2		
	R15	[It cares of individual resident]
	R28	
	R19 R23	[Evaluation system]
1	R16	
	R47 R33	[Opportunities for confrontation] [Teaching attitude of the faculty]
		[Actions for continuous quality improvement of training activity] [Professional guidance]
		[rotation plan in time]
	R2	
0	R27 R48 R6 R42 R44 R30 R41	[Overall quality of rotations]
	R31 R38 R3	
	R40	
	R26 R21	[Overall judgement] [Quality of formal lectures]
	R39	
-1	R5 R11 R22 R34 R8 R35	[Cultural activities
	R13 R4 R12	
	R17	[Regular calendar of formal lectures]
	R29 R10 R43	
	R9 R45	
-2	R36	
	R18 R46	
	R7 R20	[It poromotes clinical autonomy and reliability]
	R1	
-3		
	R24	
-4		
	R32	
-5		
-6		
-7	R37 R14

**Table 6:** **Average scores, item measures, standard errors and fit statistics of the RAC Assessment Questionnaire (measure order)**

**Item**	**Average score**	**Measure**	***SE***	**Infit**	**Outfit**
4 [It cares of individual residents]	2.8	1.54	.22	.98	.98
13 [Systems of evaluation]	2.9	1.29	.23	.95	1.01
3 [It provides opportunities of confrontation…]	3.1	.84	.23	.82	.82
12 [Quality score of the formal lessons]	3.1	.84	.23	1.41	1.35
7 [Actions to improve the quality of training]	3.2	.61	.23	.92	1.02
6[Professional guidance]	3.3	.52	.24	1.02	1.04
2[Rotation plan]	3.3	.41	.24	1.13	1.20
9 [Quality score of the rotations]	3.4	.07	.24	1.13	1.18
15 [Overall judgment]	3.6	-.50	.26	.55	.54
8 [Overall quality of the training]	3.7	-.68	.26	.72	.65
11 [Quality score of the cultural environment]	3.8	−1.10	.27	1.04	.92
1 [Formal lectures plan]	3.9	−1.48	.28	1.04	.97
10 [Autonomy and reliability]	4.1	−2.37	.29	1.16	1.13

*Note*. Greater measures indicate more difficult items (i.e., that received fewer positive evaluations).

The residents differed in severity (χ^2^(47) = 428.4, *p* < .001), but the observed agreement among them is in line with that expected (Exp = 35.8%; Obs = 36.4%). The severity of residents did not change with their gender (χ^2^(1) = 1.4, *p* = .23), whereas the differences observed among residents attending different years of program are barely sufficient (χ^2^(4) = 24.6, *p* < .001; *R* = .58).

## Discussion

This report presents the validation process of three questionnaires designed for the residents to assess,the quality of the training provided by each faculty member, the quality of the professional experience they maturated during the various rotations they have to go through and the functioning of the RAC within the context of the Paediatric Residency Program of the University of Padua. In their final form, the questionnaires produced unidimensional measures and our results suggest that they are valid and reliable evaluation tools. TAQ and RAQ allowed reliable assessments of tutors and rotations, and distinguished them into 5–6 levels of different quality. In brief, the three questionnaires provided a reliable assessment of the strengths and of the weaknesses of the tutors, the rotations, and the RAC. The agreement observed among judges was coherent to the predicted values. The usage of the questionnaires is immediate and does not require to train judges to have a shared interpretation of the items.

The MFRM represents a valuable tool for the validation of these questionnaires. Being a Rasch model, the MFRM allows transforming ordinal raw scores into interval measures. This is a relevant feature of the model since researchers showed serious concerns toward measuring ordinal raw scores as they were interval measures [29,30]. Erroneous conclusions may derive from applying parametric analyses to ordinal data [31]. In addition, the MFRM estimates judge severity and removes it from the measurement [13]. This allows the elimination of the dependence of the evaluations on the severity of judges, which is a serious concern when the different judges evaluate different persons. It is worth noting that Rasch models are especially demanding of data that satisfy the requirements for constructing measures [32]. The items removed during the validation process are an example of data that do not adequately conform to the model.

In conclusion, the data herein presented allows us to be confident, upon further internal refining, of the validity of these questionnaires for other Italian pediatric residency programs. Thus, the work presented served to enrich the armamentarium of tools the resident medical programs can use to monitor their functioning. Their larger application is now needed in order to consolidate and further refine the data herein presented. The development and validation of these tools is indeed a sort of continuous exercise. The challenge of documenting how faculty and the RAC respond to the feed-backs generated by these tools and ultimately, their impact on the quality of the training, and above all, on the quality of the pediatricians licensed by the program, remains an open issue to face with [33,34].

## Additional file

Additional file 1:
**Tutor Assessment Questionnaire; Rotation Assessment Questionnaire; Resident Affaire Committee Assessment Questionnaire.**
