# Supplementary material for: Validating a set of tools designed to assess the perceived quality of training of pediatric residency programs
Source: Ital J Pediatr. 2015 Jan 20;41:2. doi: 10.1186/s13052-014-0106-2 (PMC4339004; doi:10.1186/s13052-014-0106-2)
Supplement: Additional file 1: — Tutor Assessment Questionnaire; Rotation Assessment Questionnaire; Resident Affaire Committee Assessment Questionnaire. [file 13052_2014_106_MOESM1_ESM.docx]

Additional file 1

**Tutor Assessment Questionnaire**

The Tutor:

1. respects the timetables of the collective activities involving residents *
2. regularly gives ward lectures
3. is effective in conducting ward and frontal lectures
4. scrutinizes clinical problems with the residents
5. promotes self-education
6. reviews clinical documentation produced by residents
7. promotes autonomyand accountability
8. encourages performing invasive procedures
9. Overall judgment

**Rotation Assessment Questionnaire**

1. A clear definition of learning objectives was provided (at the beginning of the rotation)
2. The learning objectives have been met
3. It was actually organized as declared*
4. Teaching activities were regularly delivered*
5. It contributed to improve my pediatric knowledge
6. It contributed to improve my professional competence
7. It contributedto improve my clinical skills
8. It has been a useful educational experience
9. It has been a enriching experiencefrom a human point of view
10. It encouraged personal study
11. It allowed the participationin other cultural activities of the program*
12. Overall judgment

**Resident Affaire Committee Assessment Questionnaire**

1. It provides the calendar of formal lecturesin time
2. It provides the rotation plans in time
3. It promotes opportunitiesof confrontation with all the residents
4. It cares of individual resident
5. It providesresidents with individualized feedbacksregarding the evaluation they received*
6. It providesprofessional guidance
7. It implements actions for continuous quality improvement of the training activity

Please provide a numerical score to evaluate:

1. the overall quality of the training
2. the overall quality of the rotations
3. the capacity of the program to promote clinical autonomy and reliability
4. thecultural activities promoted by the program
5. thequality of the formal lectures
6. the evaluation system
7. the teaching attitude of the faculty
8. Overall judgment
